# Supplementary material for: True S-cones are concentrated in the ventral mouse retina and wired for color detection in the upper visual field
Source: eLife. 2020 May 28;9:e56840. doi: 10.7554/eLife.56840 (PMC7308094; doi:10.7554/eLife.56840)
Supplement: Supplementary file 1. — (A) Cone numbers in different retinal areas along the dorsoventral axis in pigmented and albino mouse. Three images/area (dorsal, medial and ventral) from four retinas/strain. Different cone type quantifications are shown as average ± SD, corresponding to the percentages shown in Figure 1C. The total number of cones analyzed per location and strain are shown in the last column. Total number of cones (B) or S-cone Bipolar cells (SCBCs, C) in eight retinas/mouse strain or line (average ± SD, see also Figure 2B). Significant differences between strains p<0.05 (*), p<0.01 (**), p<0.001 (***), p<0.0001 (****). [file elife-56840-supp1.docx]

**Supplementary file 1.** (A) Cone numbers in different retinal areas along the dorsoventral axis in pigmented and albino mouse. Three images/area (dorsal, medial and ventral) from four retinas/strain. Different cone type quantifications are shown as average ± SD, corresponding to the percentages shown in Fig 1C. The total number of cones analyzed per location and strain are shown in the last column. Total number of cones (B) or S-cone Bipolar cells (SCBCs, C) in eight retinas/mouse strain or line (average ± SD, see also Figure 2B). Significant differences between strains *p*<0.05 (*), *p*<0.01 (**), *p*<0.001 (***), *p*<0.0001 (****).

| A. Cones per retinal area | | | | | | | | | | | |
| --- | --- | --- | --- | --- | --- | --- | --- | --- | --- | --- | --- |
| Retinal location | | Strain | | **M^+^S^+^** | | **M^+^S^-^** | | **S^+^M^-^** | | ***Total # of* cones** | |
| Dorsal | | Pigmented | | 6.3 ± 10.5 | | 205 ± 32.4 | | 2.8 ± 1.9 | | 2,568 | |
|  |  | Albino | | 189 ± 27.5**** | | 14.8 ± 5.7**** | | 2.5 ± 0.9 | | 2,472 | |
| Medial | | Pigmented | | 274 ± 53.7 | | 0.1 ± 0.3 | | 20.2 ± 6.7 | | 3,208 | |
|  |  | Albino | | 213 ± 14.2* | | 0.9 ± 2.6 | | 14.2 ± 7.8 | | 2,731 | |
| Ventral | | Pigmented | | 196.1 ± 26.2 | | 0 ± 0 | | 96.1 ± 14.6 | | 3,506 | |
|  |  | Albino | | 195.8 ± 18 | | 0 ± 0 | | 79.8 ± 13** | | 3,307 | |
| B. Total number of cone-type population | | | | | | | | | | | |
| Strain | **All** | | M^+^ | | S^+^ | | **M^+^S^+^** | | **M^+^S^-^** | | **S^+^M^-^** |
| Pigmented | 174,826± 6,647 | | 154,617  ± 5,801 | | 109,569  ± 4,654 | | 94,926  ± 4,526 | | 63,992  ± 4,823 | | 18,608  ± 1,989 |
| Albino | 144,914***  ± 4,354 | | 129,586***  ± 4,379 | | 135,682***  ± 5,137 | | 122,063***  ± 7,669 | | 4,132***  ± 932 | | 14,366***  ± 625 |
| C. Total number of Venus^+^S-cone Bipolar cells | | | | | | | | | | | |
| Line | **Venus^+^SCBCs** | | | | | | | | | | |
| Cpne9-Venus | 11,100 ± 903 | | | | | | | | | | |
