# Supplementary material for: True S-cones are concentrated in the ventral mouse retina and wired for color detection in the upper visual field
Source: eLife. 2020 May 28;9:e56840. doi: 10.7554/eLife.56840 (PMC7308094; doi:10.7554/eLife.56840)
Supplement: Supplementary file 2. — Quantitative data are shown as mean ± SD from the average of five DT and VN retinal areas (Figure 3C). Significant differences between retinal areas, p<0.01 (**), p<0.0001 (****). [file elife-56840-supp2.docx]

**Supplementary file 2.** True S-cone terminals and Cpne9-Venus+SCBCs connectivity in dorsotemporal (DT) and ventronasal retina (VN). Quantitative data are shown as mean ± SD from the average of five DT and VN retinal areas (Figure 3C). Significant differences between retinal areas, *p*<0.01 (**), *p*<0.0001 (****).

| Cpne9-Venus Line | DT | VN |
| --- | --- | --- |
| A. True S-cones:S-cone bipolar cell | 1.92 ± 0.16 | 4.62 ± 0.26**** |
| B. S-cone bipolar cells:true S-cone | 3.81 ± 0.44** | 1.91 ± 0.24 |
