# Supplementary material for: True S-cones are concentrated in the ventral mouse retina and wired for color detection in the upper visual field
Source: eLife. 2020 May 28;9:e56840. doi: 10.7554/eLife.56840 (PMC7308094; doi:10.7554/eLife.56840)
Supplement: Supplementary file 3. — Numbers of true S-cones (A) and Cpne9-Venus+SCBCs (B) in dorsotemporal (DT) and ventronasal (VN) circular areas (1 mm diameter, Figures 3E and 4B). Quantitative data are shown as average ± SD from eight retinas/strain or line. The mean of true S-cones and Venus+SCBCs in these circular areas was used to calculate the DT:VN and true S-cone:SCBC (C) ratios. Significant differences between strains p<0.05 (*), p<0.001 (***). True S-cones and SCBCs were significant different between DT and VN retina (p<0.0001). [file elife-56840-supp3.docx]

**Supplementary file 3.** Numbers of true S-cones (A) and Cpne9-Venus^+^SCBCs (B) in dorsotemporal (DT) and ventronasal (VN) circular areas (1mm diameter, Figs 3E and 4B). Quantitative data are shown as average ± SD from eight retinas/strain or line. The mean of true S-cones and Venus^+^SCBCs in these circular areas was used to calculate the DT:VN and true S-cone:SCBC (C) ratios. Significant differences between strains *p*<0.05 (*), *p*<0.001 (***). True S-cones and SCBCs were significant different between DT and VN retina (*p*<0.0001).

| Strain/Line | DT | VN | ***DT:VN Ratio*** |
| --- | --- | --- | --- |
| A. true S-cones | | | |
| Pigmented | 115 ± 34 | 3,359 ± 437 | 1:31 |
| Albino | 82 ± 10* | 2,346 ± 405*** | 1:29 |
| B. S-cone bipolar cells | | | |
| Cpne9-Venus | 417 ± 49 | 630 ± 58 | 1:1.5 |
| C. ***true S-cone:S-cone bipolar cell Ratio*** | | | |
| Pigmented | 1:3.6 | 5.3:1 |  |
| Albino | 1:5.1 | 3.7:1 |  |
