# Supplementary material for: True S-cones are concentrated in the ventral mouse retina and wired for color detection in the upper visual field
Source: eLife. 2020 May 28;9:e56840. doi: 10.7554/eLife.56840 (PMC7308094; doi:10.7554/eLife.56840)
Supplement: Transparent reporting form [file elife-56840-transrepform.pdf]

eLIFE
1st Floor
24 Hills Road
Cambridge CB2 1JP, UK
P 01223 855340
W elifesciences.org
T @elife

eLife's transparent reporting form

We encourage authors to provide detailed information *within their submission* to facilitate the interpretation and replication of experiments. Authors can upload supporting documentation to indicate the use of appropriate reporting guidelines for health-related research (see EQUATOR Network), life science research (see the BioSharing Information Resource), or the ARRIVE guidelines for reporting work involving animal research. Where applicable, authors should refer to any relevant reporting standards documents in this form.

If you have any questions, please consult our Journal Policies and/or contact us: editorial@elifesciences.org.

Sample-size estimation

- You should state whether an appropriate sample size was computed when the study was being designed
- You should state the statistical method of sample size computation and any required assumptions
- If no explicit power analysis was used, you should describe how you decided what sample (replicate) size (number) to use

Please outline where this information can be found within the submission (e.g., sections or figure legends), or explain why this information doesn't apply to your submission:

Sample size was based on previous publications studing same cone populations (Ortín-Martínez et al., PLoS One. 2014 Jul 16;9(7):e102392, PMID: 25029531; Ortín-Martínez et al., Invest Ophthalmol Vis Sci. 2010 Jun;51(6):3171-83, PMID: 20071667). D'Agostino & Pearson normality test analysis confirmed the Gaussian distribution for the populations studied. The size effect has been calculated on-line (https://www.socscistatistics.com/effectsize/default3.aspx) and the statistic power was 1 for the majority of the analysis (detailed in the excel-row data package). Statistical significance is depicted in the figures and figures legends. The row data and statistic analysis for the different studies can be found in the excel-row data package accompanying the manuscript. Sample size: cone estimation per retinal area (Table S1A), whole-retina cone quantifications (table S1B), S-cone bipolar cells (table S1C, and material and methods 10.3.1 section), circular areas (table S2). Statistical methods are explained in 10.3.11 section of material and methods.

Replicates

- You should report how often each experiment was performed
- You should include a definition of biological versus technical replication
- The data obtained should be provided and sufficient information should be provided to indicate the number of independent biological and/or technical replicates
- If you encountered any outliers, you should describe how these were handled
- Criteria for exclusion/inclusion of data should be clearly stated
- High-throughput sequence data should be uploaded before submission, with a private link for reviewers provided (these are available from both GEO and ArrayExpress)

Please outline where this information can be found within the submission (e.g., sections or figure legends), or explain why this information doesn't apply to your submission:

8 biological specimens from each mouse strain (C57BL6, CD1) or line (Cpne9-Venus) were used to generate the different retinal distributions, specified within the tables S1 and S2. Each retina has been considered as a independent biological replicate. 200 simulations of cones were generated for each cone assignation ("distributed", "shuffled"), it is specified in the legend of Fig 4 and material and methods 10.3.9 section. No outliers were obtained in the present study.

Statistical reporting

- Statistical analysis methods should be described and justified
- Raw data should be presented in figures whenever informative to do so (typically when N per group is less than 10)
- For each experiment, you should identify the statistical tests used, exact values of N, definitions of center, methods of multiple test correction, and dispersion and precision measures (e.g., mean, median, SD, SEM, confidence intervals; and, for the major substantive results, a measure of effect size (e.g., Pearson's r, Cohen's d)
- Report exact p-values wherever possible alongside the summary statistics and 95% confidence intervals. These should be reported for all key questions and not only when the p-value is less than 0.05.

Please outline where this information can be found within the submission (e.g., sections or figure legends), or explain why this information doesn't apply to your submission:

Raw data is provided in the excel-row data package accompanying the manuscript (Figures show information processed for visual representation). Statistical metrics, dispersion measures, and methods applied to all data and algorithms are described in the text and methods section. Statistical tests applied and p-values and confidence intervals reported are provided in the excel-row data package.

(For large datasets, or papers with a very large number of statistical tests, you may upload a single table file with tests, Ns, etc., with reference to sections in the manuscript.)

Group allocation

- Indicate how samples were allocated into experimental groups (in the case of clinical studies, please specify allocation to treatment method); if randomization was used, please also state if restricted randomization was applied
- Indicate if masking was used during group allocation, data collection and/or data analysis

Please outline where this information can be found within the submission (e.g., sections or figure legends), or explain why this information doesn't apply to your submission:

Groups were allocated according to mouse strains or line, and cell or cone-type identification. The specific grouping for each figure is indicated in the corresponding text, legend, and/or methods section. Random sampling of data was done during validation of the quantitative algorithm, and is described in the Figure S1 and methods section (10.3.6).

Additional data files ("source data")

- We encourage you to upload relevant additional data files, such as numerical data that are represented as a graph in a figure, or as a summary table
- Where provided, these should be in the most useful format, and they can be uploaded as "Source data" files linked to a main figure or table
- Include model definition files including the full list of parameters used
- Include code used for data analysis (e.g., R, MatLab)
- Avoid stating that data files are "available upon request"

Please indicate the figures or tables for which source data files have been provided:

Source data has been provided in the excel-row data package for Figures 1C, 2A-C, 3D-E, 4B and Figure S1. The computational set and code (R) used for the retina and visual space retistruct (data analysis in Fig S2) has been previously developed and published as a free package (Sterratt DC et al., PLoS Comput Biol 9(2):e1002921, PMID: 23468609). We have referenced accordingly in the material and methods 10.3.10. section and in the figure legend S2.

eLife Sciences Publications, Ltd is a limited liability non-profit non-stock corporation incorporated in the State of Delaware, USA, with company number 5030732, and is registered in the UK with company number FC030576 and branch number BR015634 at the address 1st Floor, 24 Hills Road, Cambridge CB2 1JP | August 2014
